# Passenger sequences can promote interlaced dimers in a common variant of the maltose-binding protein

**DOI:** 10.1038/s41598-019-56718-y

**Published:** 2019-12-31

**Authors:** Afaque A. Momin, Umar F. Shahul Hameed, Stefan T. Arold

**Affiliations:** King Abdullah University of Science and Technology (KAUST), Computational Bioscience Research Center (CBRC), Division of Biological and Environmental Sciences and Engineering (BESE), Thuwal, 23955-6900 Saudi Arabia

**Keywords:** Biochemistry, Biological techniques, Biophysics, Biotechnology, Structural biology

## Abstract

The maltose-binding protein (MBP) is one of the most frequently used protein tags due to its capacity to stabilize, solubilize and even crystallize recombinant proteins that are fused to it. Given that MBP is thought to be a highly stable monomeric protein with known characteristics, fused passenger proteins are often studied without being cleaved from MBP. Here we report that a commonly used engineered MBP version (mutated to lower its surface entropy) can form interlaced dimers when fused to short protein sequences derived from the focal adhesion kinase (FAK) or the homologous protein tyrosine kinase 2 (PYK2). These MBP dimers still bind maltose and can interconvert with monomeric forms *in vitro* under standard conditions despite a contact surface of more than 11,000 Å^2^. We demonstrate that both the mutations in MBP and the fused protein sequences were required for dimer formation. The FAK and PYK2 sequences are less than 40% identical, monomeric, and did not show specific interactions with MBP, suggesting that a variety of sequences can promote this MBP dimerization. MBP dimerization was abrogated by reverting two of the eight mutations introduced in the engineered MBP. Our results provide an extreme example for induced reversible domain-swapping, with implications for protein folding dynamics. Our observations caution that passenger-promoted MBP dimerization might mislead experimental characterization of the fused protein sequences, but also suggest a simple mutation to stop this phenomenon.

## Introduction

The Maltose-Binding Protein (MBP) is a component of the *Escherichia coli* maltose/maltodextrin system, which regulates the uptake and catabolism of maltrodextrins as part of the chemotactic response^1,2^. MBP is encoded by the *malE* gene as a 396-residue precursor polypeptide. This precursor contains an N-terminal extension of 26 residues that acts as a signal peptide for exporting MBP into the *E. coli* periplasmic space, where it is subsequently cleaved to yield the 370-residue mature form^3^. This mechanism can be used to export recombinant proteins into the bacterial periplasm by fusing them to an MBP sequence that includes the signal peptide. Export into the periplasm can facilitate the recovery and purification of recombinant proteins, and enables the formation of disulphide bonds^4,5^.

Fusing MBP to other proteins often also greatly enhances their stability and solubility^6,7^. The exact way in which MBP stabilizes fused proteins remains unclear, but might be linked to MBP acting as a non-specific molecular chaperone that can temporarily sequester misfolded proteins. These interactions between MBP and passenger proteins would prevent aggregation of the fused sequence and might support folding, either directly in a chaperone-like manner, or indirectly by inhibiting the competing aggregation pathway^7,8^. The capacity of MBP to enhance the solubility of passenger proteins appears markedly enhanced when the passenger protein is fused to the C-terminus of MBP, rather than to its N-terminus^9^.

Because many MBP fusion proteins lose stability once cleaved from MBP, experiments to elucidate the characteristics and function of the passenger protein are often performed in the presence of MBP. MBP is known as a stable monomeric protein with well-defined ligand binding characteristics, and hence is expected not to interfere with the characterization of the passenger protein in most cases.

MBP also crystallizes easily. So much so that the first MBP crystal structure was determined in 1991 by Quiocho and colleagues to 2.3 Å resolution from data collected on a four-circle diffractometer operated with a sealed X-ray tube^10^. To date, more than 200 structures of MBPs are deposited at the Protein Data Bank (PDB). More than 100 of these are structures of MBP fused to a passenger protein^11^. Indeed, following the successful crystallization of the ectodomain of the human T cell leukaemia virus type 1 gp21 protein as an MBP fusion protein (whereas all crystallization trials of gp21 alone failed to yield suitable crystals)^12^, MBP became popular as a means to promote crystallization of proteins of interest. Subsequently, this tendency to crystallize has been further increased by an MBP version engineered to reduce surface entropy^13^(MBPeng). In addition to increasing the chances for obtaining well-diffracting crystals, the presence of MBP also provides initial phase estimates by molecular replacement (MR) methods^11^. Currently 36 structures of MBPeng are deposited in the PDB.

Here we report two passenger protein sequences that promote the formation of an intimately interlaced dimeric form of MBPeng, featuring the largest interface area observed to date for domain-swapped proteins. Identification of this characteristic of MBPeng is important because it may mislead functional assays.

## Results and Discussion

### *Design and in vitro characterization of MBP-passenger proteins*

**Figure 1:**
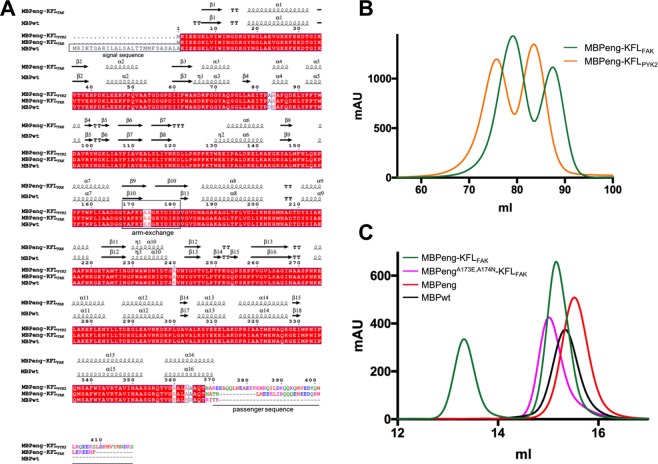
Sequences and SEC profiles of MBP constructs used. (**A**) Annotated sequence alignment was prepared using Espript 3.0 (https://espript.ibcp.fr). Passenger sequences are coloured according to hydrophobic residues: red; polar residues: green; basic residues: pink; acidic residues: blue. (**B**) SEC profile (Pharmacia S200) following large-scale purification. (**C**) Analytical SEC profile (S200 10/300). Figures were prepared using GraphPad Prism 6.0 (https://graphpad.com).

As part of our analysis of the focal adhesion kinases (FAK) and its close orthologue the protein tyrosine kinase 2 (PYK2), we were interested in testing the structural and functional properties of a short protein fragment that is part of the linker between the kinase and focal adhesion targeting (FAT) domains of these kinases. The fragments of interest being relatively short (28 and 49 residues for FAK(residues 805–832) and PYK2(residues 790–839), respectively), we decided to produce them as C-terminal MBP fusion proteins. In both proteins, this linker region was bioinformatically predicted to be helical. Therefore, we designed the constructs to be fused to MBPeng with a very short helical linker (residues Asn-Ala) as a continuation of the final C-terminal helix of MBP, according to the ‘fixed-arm carrier’ approach^13,14^ (Fig. 1A). The resulting fusion constructs were named MBPeng-KFL_FAK_ and MBPeng-KFL_PYK2_, where KFL stands for kinase-FAT linker.

Following an amylose binding column as initial purification step, we submitted the proteins to a size exclusion chromatography (SEC). During this second step, we noted the presence of two species, suggestive of monomers and dimers for both MBPeng-KFL_FAK_ and MBPeng-KFL_PYK2_ (Fig. 1B). The fact that MBP alone only eluted as a single species, with an estimated molecular weight corresponding to a monomer, suggested that the passenger proteins dimerize (Fig. 1C). However, the same KFL_FAK_ and KFL_PYK2_sequences recombinantly expressed and purified as hexa-histidine tagged proteins did not form dimers *in vitro* (Supplementary Fig. 1A,B).

### Structural analysis of the dimeric species

To understand the molecular basis for the observed dimerization, we crystallized the protein fractions corresponding to the dimeric species of MBPeng-KFL_FAK_ and of MBPeng-KFL_PYK2_ (see Methods). Both fusion proteins crystallized under several conditions. Those of MBPeng-KFL_FAK_ belonged to space group P1 and diffracted to a maximum resolution of 2.0 Å. MBPeng-KFL_PYK2_ crystals also formed in P1, however with different cell parameters, and diffracted to 3.2 Å resolution (Supplementary Table).

Structure determination by automated MR (using MoRDa wrapped in ContaMiner^15,16^) placed four and six MBP molecules in the asymmetric unit (ASU) of the MBPeng-KFL_FAK_ and MBPeng-KFL_PYK2_ crystals, respectively. The six KFL_PYK2_-fused MBP molecules were in the closed maltose-bound conformation^10^, as expected given that 2 mM maltose were included in all purification and crystallization buffers. Conversely, KFL_FAK_-fused MBP molecules were in the open domain conformation, associated with a ligand-free state^17^, and none of the four MBP active sites in the asymmetric unit showed clear electron density for maltose. These crystals grew only after 2–3 weeks, suggesting that maltose had been broken down by contaminants (enzymes or microbes), or that the crystals grew from a minority population of maltose-free molecules.

**Figure 2:**
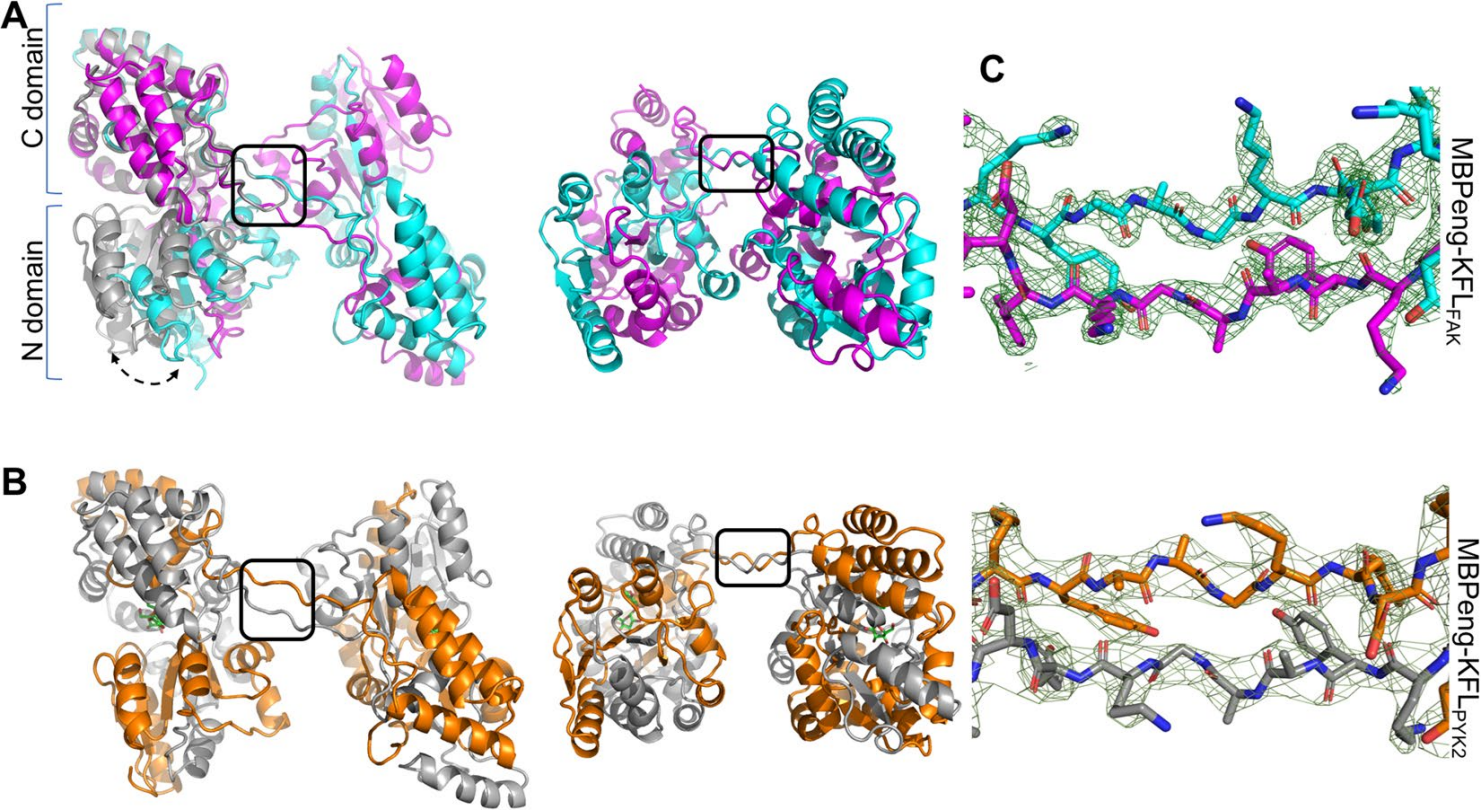
Structural analysis of interlaced MBP dimers. (**A**) 90° views of maltose-free MBPeng-KFL_FAK_. Chains are coloured in magenta and cyan. The region of arm-exchange is boxed. A monomeric MBP in its maltose-bound closed state is superimposed (grey, PDB accession 3woa). The double-headed arrow indicates the domain-movement associated with open and closed MBP forms. (**B**) Maltose-bound MBPeng-KFL_PYK2_ is shown in grey (chain A) and orange (chain B). (**C**) Omit maps (green mesh) showing the arm-exchange region (residues 172–177) for MBPeng-KFL_FAK_ (top) and MBPeng-KFL_PYK2_ (bottom). Figures were prepared using PyMol 1.8.6.2 (https://pymol.org).

During model rebuilding and refinement, it became apparent that adjacent MBP molecules formed the same intricately interlaced arm-exchange dimers in both MBPeng-KFL_FAK_ and MBPeng-KFL_PYK2_ crystals, (harbouring two and three dimers per ASU, respectively) (Fig. 2A,B). Instead of a tight β-turn at residues 173–176, the protein chains adopted an extended β-strand structure, crossing straight into the second MBP structure for both MBPeng-FAK_KFL_ and MBPeng-KFL_PYK2_ (Fig. 2C). This β-strand pairing is stabilized by six intermolecular backbone hydrogen bonds between residues 171 and 178 of both chains. This network is akin to monomeric MBPeng (e.g. PDB id 5aq9) where one single chain forms three intramolecular H-bonds. Because MBP’s polypeptide chain goes back and forth between its N-terminal and C-terminal lobes, this chain crossing resulted in highly intertwined dimers, where the two domains of both MBP molecules are constituted by both polypeptide chains (Fig. 2A,B). This structural architecture produced an extremely high contact surface between both chains (~11,300 Å^2^), corresponding to a calculated solvation free energy gain (∆^i^G) of −174.5 kcal/mol. These values are substantially larger than those of other known domain-swapped dimers (Table 1). Except for the hinge-region, the domain swapping did not significantly alter the structure of domain-swapped MBPeng compared to its canonical monomeric forms (Cα root-mean-square deviation, RMSD, of 0.40–0.42 Å over 365–370 residues, and RMSD of 0.50–0.70 Å over 366–375 residues for the nine most similar maltose-bound and apo forms, respectively; Supplementary Fig. 2).

**Table 1:** Selected examples of known domains-swapped protein structures.

	interface area, Å^2^	Δ^i^G kcal/mol	hydrogen bonds	salt bridges	di-sulphide bonds
*MBPeng-KFL*_*FAK*_	11228	−174.5	159	7	0
*MBPeng-KFL*_*PYK2*_	11322	−164.5	177	7	0
*LeuA (1sr9)*	7646	−53.1	99	39	0
*prion (1i4m)*	3119	−57.3	28	6	2
*cystatin C*_*a*_ *(1tij)*	3096	−39.2	58	13	0
*cystatin C*_*d*_ *(1g96)*	2844	−38.4	50	10	0
*suc1 (1sce)*	2163	−26.8	30	6	0
*RNaseA (1a2w)*	1932	−14.3	35	3	0
*GB1 (1Q10)*	1762	−33.5	18	0	0

Values were calculated by the ‘Protein interfaces, surfaces and assemblies service PISA at the European Bioinformatics Institute^32^. ΔiG: calculated solvation free energy gain upon formation of the interface.

LeuA: Mycobacterium tuberculosis LeuA.

hPrion: domain-swapped dimer of the human prion protein.

cystatin C_a_: 3D domain-swapped human cystatin C with amyloid-like intermolecular beta-sheets.

cystatin C_d_: 3D domain-swapped dimeric human cystatin C.

Suc1: domain-swapped dimer of the cell cycle-regulatory protein suc1.

RNaseA: N-terminal domain-swapped dimer of bovine RNase A.

GB1: domain-swapped dimeric mutant of the B1 domain of Streptococcal protein G.

Following structural refinement, clear electron density was only found for the Asn-Ala linker and the first (in the KFL_PYK2_ crystal) or the first five (KFL_FAK_) passenger protein residues. Mass spectrometric analysis on harvested and washed protein crystals of MBPeng-FAK_KFL_ and MBPeng-KFL_PYK2_ produced an experimental mass (43,843 ± 2 Da and 46137 ± 2 Da) matching the calculated mass for these constructs (43807 Da and 46220 Da for MBPeng-FAK_KFL_ and MBPeng-KFL_PYK2_, respectively). Therefore, the KFL_FAK_ and KFL_PYK2_ sequences were present in the crystals, but are too mobile to produce observable electron density.

**Figure 3:**
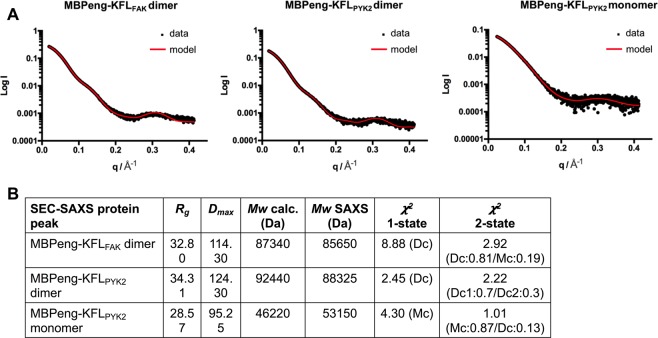
SAXS analysis. (**A**) SAXS scattering pattern (black) and fitted scattering pattern calculated from best-fitting two-state model (red). ‘Dimer’ and ‘monomer’ refer to the corresponding peaks of the SEC-SAXS experiment (see Supplementary Fig. 1C,D). (**B**) Table showing SAXS-derived radius of gyration (Rg), maximum diameter (Dm), calculated and SAXS-derived molecular weight (Mw), and the fitting parameters (𝛘^2^) for the single-state (1-state) and two-state (2-state) models. Best-fitting models were selected from a pool of models containing five representatives of each, closed maltose-bound interlaced MBP-fusion dimers (Dc), open ligand-free dimers (Do), closed monomers (Mc) and open monomers (Mo). For each type of model, the five representatives differ by the positioning of the KFL_FAK_ or KFL_PYK2_ sequence. The type of selected best scoring model is indicated. For the 2-state models, the relative contribution of each individual model is given as % value. Figures were prepared using GraphPad Prism 6.0 (https://graphpad.com).

Small-angle X-ray scattering (SAXS) pattern calculated for models based on the crystallographic interlaced MBP dimers (where the passenger sequences were assumed to be mobile) fitted the experimental size-exclusion chromatography–fed small angle X-ray scattering (SEC-SAXS) data very well (Fig. 3, Supplementary Fig. 1C,D). The SEC-SAXS buffer contained 2 mM maltose, and scattering pattern for both KFL_FAK_ and KFL_PYK2_ sequences were best fitted by MBP molecules in their closed, maltose bound conformation (Fig. 3B). Hence, the interlaced MBP dimers were already present under normal buffer conditions, and did not only form under the crystallization conditions used.

### The interlaced dimers are specific to MBPeng and the fused sequences

**Figure 4:**
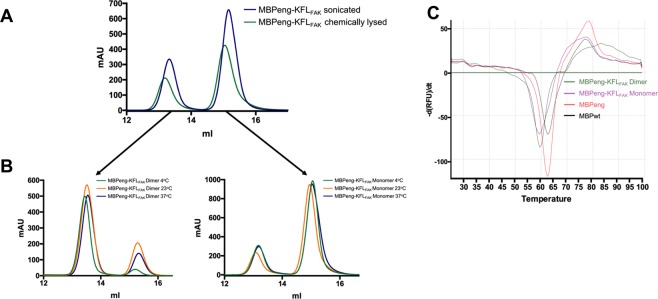
Stability and exchange rate of MBPeng monomers and dimers in solution. (**A**) Analytical SEC on MBPeng-KFL_FAK_ obtained following cell lysis by sonication or chemical lysis. (**B**) Central fractions of the monomeric or dimeric peaks from (**A**) were incubated for one week at different temperatures and then subjected to analytical SEC. (**C**) Thermal stability assay (DSF) showing the first derivative of melting curves. Figures were prepared using GraphPad Prism 6.0 (https://graphpad.com).

The high degree of MBP polypeptide chain interlacing suggested that these dimers resulted from refolding under high protein concentrations (as opposed to partial opening of a dynamic 3D structure; see^18^ for an example related to FAK). We reasoned that such correlated (un)folding might result from short local overheating during sonication of bacterial cells. However, protein purification of MBPeng-KFL_FAK_ without sonication (using a chemical protein extraction protocol) produced the same SEC profile showing monomers and dimers (Fig. 4A), demonstrating that overheating was not needed. Following incubation of monomeric or dimeric fractions for one week at 37 °C, we observed that about 20% of the molecules had converted into dimers and monomers, respectively (Fig. 4B). Conversion of the dimers to monomers was markedly decreased at 4 °C, whereas temperature did not significantly affect the monomer-to-dimer conversion rate. We concluded that monomers and dimers exchange under standard buffer concentrations.

Due to the intimacy of chain intertwining, MBPeng molecules must almost completely unfold in order to interconvert between monomers and dimers. The C-terminal helix and linker sequence contained MBPeng-specific substitutions (K363A, D364A, I369A), which destabilize the interaction of this region with the core of the protein (through charge complementarity with D185, H-bond to Q356, and hydrophobic interactions, respectively) possibly reducing the overall protein stability. Therefore, we next investigated if our MBPeng fusion constructs had a reduced thermal stability. The melting temperature (*Tm*) of MBPeng (without passenger sequences) was only slightly lower compared to MBPwt (61.8 ± 0.39 °C and 62.5 ± 0.28 °C, respectively) (Fig. 4C). The *Tm* of MBPeng-KFL_FAK_ monomer (60.1 ± 0.43 °C) and dimer (59.0 ± 0.49 °C) were lower than MBPeng alone, demonstrating that these particular passenger sequences further destabilized the fusion protein. Accordingly, MBPeng alone did not produce dimers in SEC (Fig. 1c). We concluded that both MBPeng and the particular passenger sequences were necessary to produce the interlaced MBP dimers.

We noted that the arm-exchange hinge region contained MBPeng-specific substitutions (E173A, N174A) compared to wild-type MBP (MBPwt) **(**Fig. 1A**)**. In the monomeric maltose-bound and apo forms of MBPwt, both residues are within the most favoured regions of the Ramachandran plot, and are exposed to solvent, without engaging intramolecular interactions (see, for example, PDB entries 3woa and 1ziu). However, the corresponding A173 and A174 in MBPeng monomers are also in the most favoured regions (e.g. PDB 4egc), showing that the substitution does neither lead to a loss of stabilising contacts, nor introduces significant strain in the loop. To investigate if the E173A/N174A mutations nonetheless contributed to arm-exchange, we mutated them back into the wild-type glutamic acid and asparagine. MBPeng^A173E,A174N^-KFL_FAK_ produced only monomeric SEC peaks, demonstrating that reversing the two mutations was sufficient to block domain-swapped dimers (Fig. 1C). The *Tm* of MBPeng^A173E,A174N^-KFL_FAK_ (62.1 ± 0.34 °C) was also increased by 2 °C compared to the monomeric MBPeng, demonstrating a loss of stability associated with the E173A/N174A mutations (Supplementary Figure 1E). In MBPwt, E173 is part of a charge-charge network of this loop region, which is lost in MBPeng. Thus, we identified the double substitution E173A/N174A in the loop region as a key driver for the formation of interlaced MBPeng dimers.

## Discussion and Conclusion

MBP is arguably one of the most highly used and best characterized protein tags. Therefore, it was surprising that two passenger protein sequences promote the formation of highly interwoven dimers in a commonly used MBP form engineered to enhance crystallization (MBPeng^13^).

The presence of 3D domain swapping has been noted in ~60 protein structures to date, including engineered and naturally occurring examples with biological functions (e.g ^19–21^. These structures have provided insights into protein folding, multimerization and evolution^22^. Compared to the known examples, the interlaced MBP structures we present herein are unusual because of their extremely large surface area involved, but also the driving force for their domain exchange appears atypical. Domain swapping is commonly promoted by the alteration of the hinge loop length, strain or flexibility, and often involves proline or glycine residues^22^. However, intriguingly, none of these mechanisms appears to explain domain-swapping in our case. Moreover, the domain-swapped MBPeng dimer is actually less stable than the MBPeng monomer (∆*Tm* = 1 °C), and *in silico* modelling showed that there are no steric clashes or charge-charge repulsions that would prevent MBPwt from adapting the extended hinge region conformation of the domain-swapped MBPeng. It is further unlikely that MBP domain-swapping was linked to stalling of the translation process, because both sequences were codon optimized for *E. coli* expression, and we also observed monomer-dimer conversion *in vitro*.

Rather, the swapping mechanism might involve the electro-statics of the hinge-loop, which contains closely located negative (E173, D178, D181) and positive (K171, K176, K180) charges. These charges are interspaced by hydrophobic residues (F170, Y172, Y177, I179, V182) that pin the loop to the protein surface and expose all the charged residues to the opposite side of the loop. In MBPwt, charge complementarity not only enhances protein stability, but might also favour that the polypeptide chain folds back onto itself. With an imbalanced charge ratio, as present in MBPeng, chain back-folding might be delayed, allowing domain-exchanged dimers to form.

In addition to hinge loop mutations, domain-swapping also required the presence of specific passenger sequences. In MBPwt, the presence of the N-terminal signal peptide slows down the protein folding rate at least 5-fold^23^. Although our sequences were C-terminally fused to MBP, a similar, yet sequence-specific mechanism might also slow down folding rates of our constructs, promoting concerted interlaced refolding of our sequences. Although the KFL_FAK_ and KFL_PYK2_ sequences are only 40% identical and of different length (28 and 49 residues, respectively), both share characteristics that might be at the origin of their capacity to promote MBP dimers: both possess a similarly low theoretical pI (4.71 and 4.99, for KFL_FAK_ and KFL_PYK2_, respectively) and contain the sequence pattern Q-Q-[QERK](2)-M-X-[ED](2)-X(2)-W-L-X(2)-E(2)-[RK] (Fig. 1A). This pattern is well conserved across FAK and PYK2 sequences, however it is unknown if it has a particular biological function. Conversely, we found no indication to suggest that the KFL_FAK_ and KFL_PYK2_ sequences act through strongly associating with regions of MBPeng.

The conserved FAK/PYK2 pattern is not present in any of the passenger protein sequences of the 36 MBPeng structures currently deposited in the PDB, and none of these structures showed a crystal packing that could indicate an interlaced MBP dimer as observed by us. The probability for a passenger sequence to promote MBPeng domain-exchange may therefore be low. We note, however, that the absence of crystallized MBP dimers in the PDB does not necessarily preclude the occurrence of such dimers *in vitro*, because SEC purification might have favoured monomeric over dimeric forms, and dimeric forms might not crystallize equally well because of the flexibility of the hinge region. Moreover, the presence of the KFL_FAK_ sequence only lowered the MBPeng *Tm* by ~2 °C, a reduction which might also be achieved by other passenger sequences. Although none of the other MBPeng sequences in the PDB showed dimerization, we found strong evidence for the same domain-swapped dimers in a structure of the *Salmonella enterica* sugar-binding protein MalE (PDB id 6l3e; Supplementary Fig. 3). MalE is a close homologue of *E. coli* MBP (94.32% sequence identity; Supplementary Fig. 4), and with an RMSD of 0.35 Å over 365 residues MalE was the closest structural match in the PDB to our dimer-swapped MBPeng-KFL_PYK2_. The MalE structure has not yet been published. But given that it is modelled as monomer, it is likely that the authors have not noted the domain-swapping. The MalE hinge-region sequence is identical to MBPwt, containing E173/N174 (Supplementary Fig. 4), however the side chains of E173 and K176 are invisible after the Cβ atom (Supplementary Fig. 3C). Hence, the dimer-swapping was probably promoted by a different mutation elsewhere in the protein, although a mutation in the protein sequence of either E173 and K176 cannot be ruled out.

We demonstrated that the dimeric MBP form can still bind to maltose, and that it can slowly exchange with monomeric forms *in vitro* under standard buffer conditions. Given that MBP dimerization has never been reported, these features can easily mislead investigators into believing that their passenger sequence dimerizes. Additionally, the unsuspected MBP-promoted dimerization of passenger proteins may mislead many other types of experiments, such as *in vitro* affinity measurements or cell-based assays (e.g. monomeric vs dimeric transcription factors). Hence, our observations caution that unsuspected passenger-promoted MBP dimerization might mislead experimental investigations of proteins fused to MBPeng, or possibly other MBP variants. We propose to use the reverse mutation A173E/A174N in the arm-exchange linker to rule out the occurrence of this phenomenon in MBPeng while still preserving most of the benefits of the surface entropy reduction.

## Materials and Methods

### Protein cloning, expression and purification

MBPeng-KFL_FAK_, MBPeng-KFL_PYK2_ and MBPeng were cloned by TWIST bioscience Ltd. as MBP fusion in a pJEx411c vector with kanamycin resistance. MBPwt was cloned into the pETduet-1 vector with ampicillin resistance. Transformed *E. coli* BL21(DE3) competent cells were grown at 37 °C in LB medium containing 50 µg/ml kanamycin for MBPeng-KFL_FAK_, MBPeng-KFL_PYK2_ and MBPeng and 100 µg/ml ampicillin for MBPwt protein. As the cell density reached an absorbance at 600 nm of 0.7 to 0.8, protein expression was induced with 0.25 mM IPTG for 18 h at 20 °C. Cells were then harvested, centrifuged, and the cell pellet was resuspended in binding buffer A: 75 mM Tris-HCl buffer (pH 7.5), 200 mM NaCl, 2 mM DTT, a tablet of protease inhibitor/L and 0.05% Triton X-100. The cell suspension was lysed by sonication on ice. Alternatively, chemical cell lysis was achieved through BugBuster cell lysis. The supernatant was loaded onto an amylose (NEB) column and incubated for 2 hours at 4 °C. The column was washed using binding buffer A for 5 column volumes. The MBP fusion protein was eluted with 20 mM maltose added in buffer A. The purified MBP fusion protein at 15 mg/ml was applied to a Superdex 200 column (GE Healthcare) equilibrated with 20 mM HEPES (pH 7.5), 150 mM NaCl, 2 mM Maltose and 2 mM DTT. MBP fusion protein was eluted as a double peak describing as monomer and dimer species present in the solution. The monomer and dimer peaks were collected separately and were concentrated using ultrafiltration membrane (Merck Millipore) with 10 kD MWCO for experiments and crystallization trials.

The gene fragments for 6xHis-KFL_FAK_ and 6xHis-KFL_PYK2_ were cloned using pET32a modified expression plasmid. Plasmids were transformed into *E. coli* BL21(DE3) and expressed as described above for the MBP fusion proteins. Cells were harvested and the cell pellet was resuspended in buffer A: 75 mM Tris (pH 8.0), 500 mM NaCl, 1 mM DTT, 1 tablet protease inhibitor/L, and 0.05% TritonX-100. The cell suspension was lysed by sonication on ice. After cell lysis and centrifugation, the protein was purified from supernatant using a 5 ml HisTrap column (GE Healthcare). Weakly bound proteins and contaminants were washed off using buffer A complemented with 10 mM imidazole. The proteins were eluted using 500 mM imidazole. The fragments were further purified using a HiLoad 26/60 Superdex 75 column (GE Healthcare) with buffer containing 20 mM HEPES, pH 7.5, 150 mM NaCl and 2 mM DTT. The proteins were concentrated using ultrafiltration membrane (Merck Millipore) with 3 kD MW cut-off for experiments

### Protein crystallization

The fractions containing dimeric species for MBPeng-KFL_FAK_ and MBPeng-KFL_PYK2_ were used for crystallization. Crystals were obtained with the hanging drop vapor diffusion method. MBPeng-KFL_FAK_ crystals were obtained in two weeks by equilibrating 1.0 μl of protein (15 mg/ml) mixed with 1.0 μl of reservoir solution (2 M Ammonium sulfate, 0.1 M CAPS/Sodium hydroxide pH 10.5 and 0.2 M Lithium sulfate) at 23 °C. Crystals for MBPeng-KFL_PYK2_ grew within three 3 days by equilibrating 1.0 μl of protein (15 mg/ml) mixed with 1.0 μl of reservoir solution (30% (v/v) PEG 400 and 0.1 M CAPS/Sodium hydroxide pH 10.5 at 23 °C. 25% glycerol was added to the well solution as a cryo‐protectant, and the crystals were flash‐cooled in liquid nitrogen. Data for MBPeng-KFL_FAK_ and MBPeng-KFL_PYK2_ and were collected at 100 K at the beamline Proxima 2 A at the SOLEIL Synchrotron (France), using an EIGER 6 M detector (proposal numbers 20181104 and 20180576)^24^. The data were processed in XDS.^25^.

### Protein structure determination

Initial phases were obtained by ContaMiner^16^, based on the MoRDa MR pipeline^15^. Automated rebuilding by Buccaneer^26^ was followed by iterative manual rebuilding in Coot^27^ and automated refinement with Phenix^28^. The model was evaluated using MolProbity^29^.

### Small angle X-ray scattering

SEC-SAXS data were recorded at the SWING beamline (SOLEIL, Saint-Aubin, France) at λ = 1.03 Å. The detector-sample distance was 1.8 m, resulting in the momentum transfer range of 0.01 Å-1 < q < 0.5 Å-1. Buffer scattering contributions were calculated from the buffer (20 mM HEPES, 150 mM NaCl, 2 mM DTT and 2 mM Maltose) eluted before proteins, and subtracted from the protein scattering intensity using SWING’s on-site FOXTROT software. Data were analysed using PRIMUS, BUNCH, DAMMIN, DAMMIF and DAMAVER of the ATSAS software package^30^ and FOXS^31^.

### Analytical size exclusion chromatography

SEC Experiments were performed using a Superdex 200 30/300 column (GE healthcare) on an AKTA pure. SEC was performed at 6 mg/ml protein concentration in an identical buffer of 20 mM HEPES, 150 mM NaCl, 2 mM DTT and 2 mM Maltose for MBPeng-KFL_FAK_, MBPeng^A173E,A174N^-KFL_FAK_, MBPwt and MBPeng.

### Differential scanning fluorimetry

DSF experiments were performed in 20 mM HEPES (pH 7.5), 150 mM NaCl, 2 mM DTT and 2 mM Maltose. MBPeng-KFL_FAK_, MBPeng-KFL_PYK2_, MBPeng^A173E,A174N^-KFL_FAK_, MBPeng, MBPwt and the fluorescent dye SYPRO Orange were used at a concentration of 10 μM. The samples in triplicates were heated from 25 °C to 99 °C at a rate of 0.03 °C/s on a CFX96 Real Time PCR system (BioRad). The melting temperature (*Tm*) was estimated by least-squares fitting of a generalized sigmoid, and the inflection point was computed using BioRad software.

### Microscale thermophoresis

The proteins were serially diluted starting from 200 μM (KFL_FAK_) and 200 μM (KFL_PYK2_) in reaction buffer (20 mM HEPES pH 7.5, 150 mM NaCl, 1 mM DTT, 0.05% Tween-20). 6xHistag-KFL_FAK_ and 6xHistag-KFL_PYK2_proteins were labelled using the HisTag Ni-NTA dye at 0.1 μM. The final concentration of the labelled protein was kept at 0.05 μM. The experiment was performed in biological triplicates at 50% LED power and medium MST power detected by the machine in MST Premium Capillaries on a Monolith NT.115 device at 25 °C (NanoTemper Technologies). Data were analysed using company provided NT Analysis software.

### Mass-spectrometry analysis

MBPeng-KFL_FAK_ and MBPeng-KFL_PYK2_ crystals were thoroughly washed with the crystallisation buffer. High-performance liquid chromatography (HPLC) analysis was performed using UltiMate 3000 UHPLC System. The chromatographic separation was carried out on Phenomenex Analytical C4 column (Aeris 3.6 µm WIDEPORE, 200 Å, LC Column 100 × 2.1 mm). The mobile phases consisted of solvent A 0.1% formic acid in water and solvent B 0.1% formic acid in acetonitrile. The protein was eluted using a linear gradient; solvent B was increased from 5% B at t = 1 min to 80% B at t = 14 minute. This concentration of buffer B was maintained for 4 minutes (t = 14 to t = 18), at t = 18.5 min the column was equilibrated with 95% of buffer A for 6.5 minutes. The Maxis QTOF mass spectrometer (Bruker Daltonics, Bremen, Germany) operating in positive ion mode, the electrospray process was initiated using a voltage of 4200 V. The mass was calibrated with Cytochrome C at the beginning of every run delivering a mass accuracy of <2ppm. Data were acquired automatically under the control of Hystar using a TOF MS acquisition rate of 3 Hz over the mass range of 400–3000 m/z. The electrospray interface settings were the following: nebulizer pressure 4 bar, drying gas 8 L/min, 220 °C. The data were analysed using Bruker Compass Data Analysis 4.0.

## Supplementary information

Supplementary Information.

## Data Availability

Atomic coordinates for MBPeng-KFL_FAK_ and MBPeng-KFL_PYK2_ have been deposited in the Protein Data Bank with accession codes 6LES and 6LF3 respectively.

## References

[1] Higgins CF (1992). ABC transporters: from microorganisms to man. Annu. Rev. Cell Biol..

[2] Chen J (2013). Molecular mechanism of the *Escherichia coli* maltose transporter. Curr. Opin. Struct. Biol..

[3] Kellermann O, Szmelcman S (1974). Active transport of maltose in *Escherichia coli* K12. Involvement of a “periplasmic” maltose binding protein. Eur. J. Biochem..

[4] Bedouelle H, Duplay P (1988). Production in *Escherichia coli* and one-step purification of bifunctional hybrid proteins which bind maltose. Export of the Klenow polymerase into the periplasmic space. Eur. J. Biochem..

[5] Malik A (2016). Protein fusion tags for efficient expression and purification of recombinant proteins in the periplasmic space of *E. coli*. 3 Biotech..

[6] LaVallie ER (1993). A thioredoxin gene fusion expression system that circumvents inclusion body formation in the E. coli cytoplasm. Biotechnol. (N. Y.).

[7] Kapust RB, Waugh DS (1999). *Escherichia coli* maltose-binding protein is uncommonly effective at promoting the solubility of polypeptides to which it is fused. Protein Sci..

[8] Nallamsetty S, Waugh DS (2006). Solubility-enhancing proteins MBP and NusA play a passive role in the folding of their fusion partners. Protein Expr. Purif..

[9] Raran-Kurussi S, Keefe K, Waugh DS (2015). Positional effects of fusion partners on the yield and solubility of MBP fusion proteins. Protein Expr. Purif..

[10] Spurlino JC, Lu GY, Quiocho FA (1991). The 2.3-A resolution structure of the maltose- or maltodextrin-binding protein, a primary receptor of bacterial active transport and chemotaxis. J. Biol. Chem..

[11] Waugh DS (2016). Crystal structures of MBP fusion proteins. Protein Sci..

[12] Kobe B, Center RJ, Kemp BE, Poumbourios P (1999). Crystal structure of human T cell leukemia virus type 1 gp21 ectodomain crystallized as a maltose-binding protein chimera reveals structural evolution of retroviral transmembrane proteins. Proc. Natl Acad. Sci. U S Am..

[13] Moon AF, Mueller GA, Zhong X, Pedersen LC (2010). A synergistic approach to protein crystallization: combination of a fixed-arm carrier with surface entropy reduction. Protein Sci..

[14] Jin T (2017). Design of an expression system to enhance MBP-mediated crystallization. Sci. Rep..

[15] Vagin A, Lebedev A (2015). MoRDa, an automatic molecular replacement pipeline. Acta Cryst..

[16] Hungler A, Momin A, Diederichs K, Arold ST (2016). ContaMiner and ContaBase: a webserver and database for early identification of unwantedly crystallized protein contaminants. J. Appl. Crystallogr..

[17] Sharff AJ, Rodseth LE, Spurlino JC, Quiocho FA (1992). Crystallographic evidence of a large ligand-induced hinge-twist motion between the two domains of the maltodextrin binding protein involved in active transport and chemotaxis. Biochemistry.

[18] Arold ST, Hoellerer MK, Noble ME (2002). The structural basis of localization and signaling by the focal adhesion targeting domain. Structure.

[19] Bourne Y (1995). Crystal structure of the cell cycle-regulatory protein suc1 reveals a beta-hinge conformational switch. Proc. Natl Acad. Sci. U S Am..

[20] Kadare G (2015). Conformational dynamics of the focal adhesion targeting domain control specific functions of focal adhesion kinase in cells. J. Biol. Chem..

[21] Qu C (2000). 3D domain swapping modulates the stability of members of an icosahedral virus group. Structure.

[22] Rousseau F, Schymkowitz J, Itzhaki LS (2012). Implications of 3D domain swapping for protein folding, misfolding and function. Adv. Exp. Med. Biol..

[23] Beena K, Udgaonkar JB, Varadarajan R (2004). Effect of signal peptide on the stability and folding kinetics of maltose binding protein. Biochemistry.

[24] Shahul Hameed, U. *et al*. Structural basis for specific inhibition of the highly sensitive ShHTL7 receptor. *EMBO reports* **19**, 10.15252/embr.201745619 (2018). 10.15252/embr.201745619 PMC6123649 30021834

[25] Kabsch W (2010). Xds. Acta Crystallogr. D. Biol. Crystallogr.

[26] Cowtan K (2006). The Buccaneer software for automated model building. 1. Tracing protein chains. Acta Crystallogr. D. Biol. Crystallogr.

[27] Emsley P, Lohkamp B, Scott WG, Cowtan K (2010). Features and development of Coot. Acta Crystallogr. D. Biol. Crystallogr.

[28] Adams PD (2013). Advances, interactions, and future developments in the CNS, Phenix, and Rosetta structural biology software systems. Annu. Rev. biophysics.

[29] Davis IW (2007). MolProbity: all-atom contacts and structure validation for proteins and nucleic acids. Nucleic acids Res..

[30] Franke D (2017). ATSAS 2.8: a comprehensive data analysis suite for small-angle scattering from macromolecular solutions. J. Appl. Crystallogr..

[31] Schneidman-Duhovny D, Hammel M, Tainer JA, Sali A (2016). FoXS, FoXSDock and MultiFoXS: Single-state and multi-state structural modeling of proteins and their complexes based on SAXS profiles. Nucleic acids Res..

[32] Krissinel E, Henrick K (2007). Inference of Macromolecular Assemblies from Crystalline State. Journal of molecular biology.

