# Supplementary material for: Passenger sequences can promote interlaced dimers in a common variant of the maltose-binding protein
Source: Sci Rep. 2019 Dec 31;9:20396. doi: 10.1038/s41598-019-56718-y (PMC6938514; doi:10.1038/s41598-019-56718-y)
Supplement: Supplementary file 1 — Supplementary Information. [file 41598_2019_56718_MOESM1_ESM.docx]

**SUPPLEMENTARY MATERIAL FOR**

**Passenger sequences can promote interlaced dimers in a common variant of the maltose-binding protein**

Afaque A. Momin, Umar F. Shahul Hameed, Stefan T. Arold*

King Abdullah University of Science and Technology (KAUST), Computational Bioscience Research Center (CBRC), Division of Computer, Electrical and Mathematical Sciences & Engineering (CEMSE), Thuwal 23955-6900, Saudi Arabia

**SUPPLEMENTARY TABLE 1**

|  | **MBPeng-KFL_FAK_** | **MBPeng-KFL_PYK2_** |
| --- | --- | --- |
| **Wavelength (Å)** | 0.98 | 0.98 |
| **Resolution range** | 47.45 - 2.0 (2.08 - 2.00) | 47.86 - 3.205 (3.32 - 3.21) |
| **Space group** | P 1 | P 1 |
| **Unit cell** | 65.91 71.96 83.39 97.84 90.03 106.55 | 78.78 92.74 93.61 112.46 101.66 94.93 |
| **Total reflections** | 328157 (28311) | 115688 (10238) |
| **Unique reflections** | 93102 (8178) | 37579 (3539) |
| **Multiplicity** | 3.5 (3.5) | 3.1 (2.9) |
| **Completeness (%)** | 95.33 (84.49) | 96.78 (90.43) |
| **Mean I/sigma(I)** | 8.12 (1.20) | 7.44 (1.03) |
| **Wilson B-factor** | 39.84 | 100.77 |
| **R-merge** | 0.1033 (1.178) | 0.1046 (0.8408) |
| **R-meas** | 0.1229 (1.386) | 0.1257 (1.019) |
| **R-pim** | 0.06574 (0.7233) | 0.06884 (0.5674) |
| **CC1/2** | 0.995 (0.6) | 0.996 (0.56) |
| **CC*** | 0.999 (0.866) | 0.999 (0.847) |
| **Reflections used in refinement** | 93016 (8172) | 37495 (3524) |
| **Reflections used for R-free** | 4653 (409) | 1862 (174) |
| **R-work** | 0.2041 (0.3641) | 0.2439 (0.3975) |
| **R-free** | 0.2310 (0.3680) | 0.2838 (0.4046) |
| **CC(work)** | 0.957 (0.728) | 0.878 (0.327) |
| **CC(free)** | 0.951 (0.756) | 0.857 (0.350) |
| **Nr. of non-hydrogen atoms** | 11755 | 17148 |
| **macromolecules** | 11317 | 17010 |
| **ligands** | 10 | 138 |
| **solvent** | 428 |  |
| **Protein residues** | 1472 | 2216 |
| **RMS(bonds)** | 0.010 | 0.009 |
| **RMS(angles)** | 1.27 | 1.53 |
| **Ramachandran favored (%)** | 98.84 | 98.45 |
| **Ramachandran allowed (%)** | 1.16 | 1.36 |
| **Ramachandran outliers (%)** | 0.00 | 0.18 |
| **Rotamer outliers (%)** | 1.40 | 4.64 |
| **Clashscore** | 3.20 | 9.96 |
| **Average B-factor** | 50.33 | 55.01 |
| **macromolecules** | 50.40 | 54.63 |
| **ligands** | 83.57 | 101.60 |
| **solvent** | 47.61 |  |
| **Number of TLS groups** | 20 | 12 |

Statistics for the highest-resolution shell are shown in parentheses.


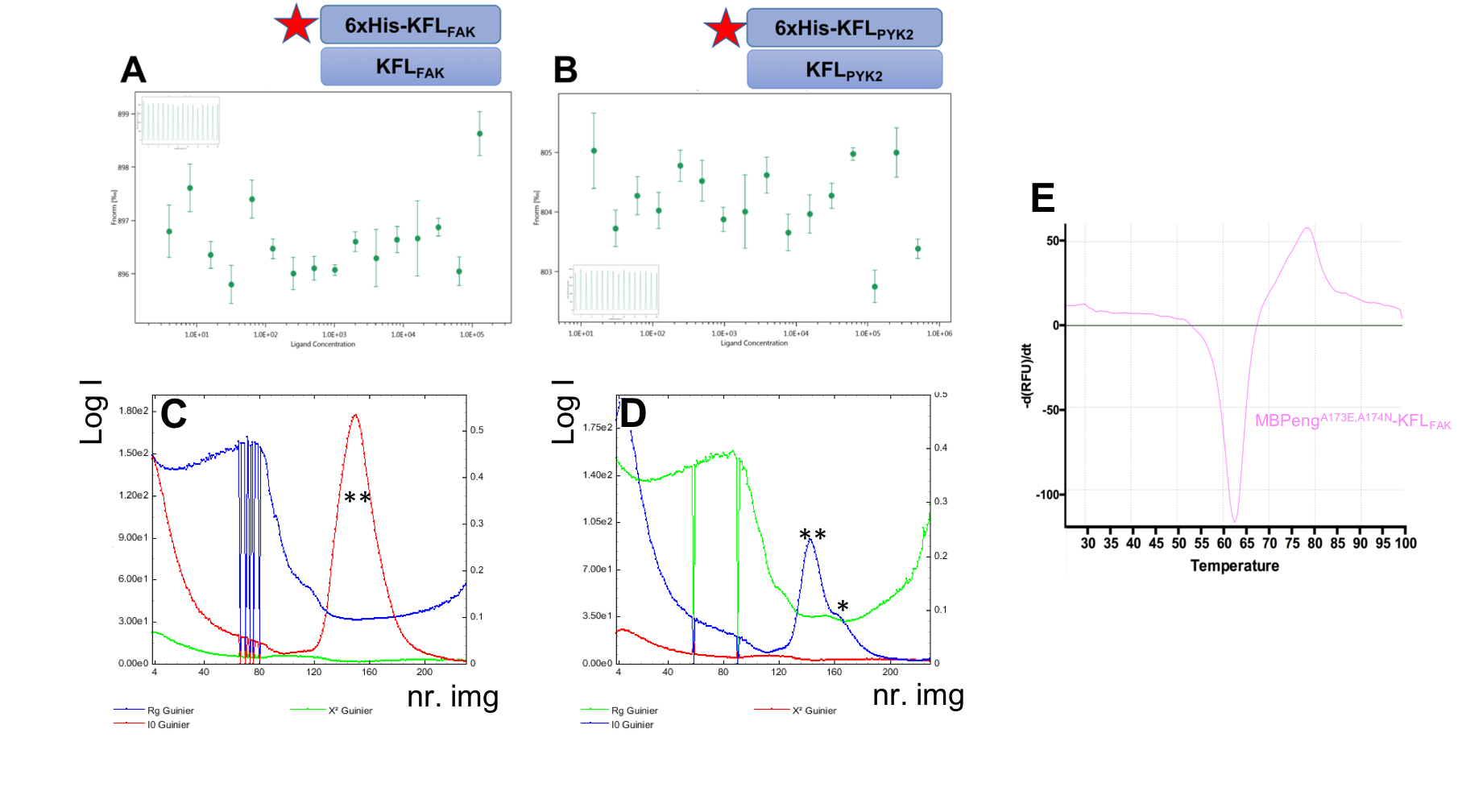


**Supplementary Figure 1. (A,B)** Microscale Thermophoresis (MST) dimerization experiment where fluorescently labelled 6xHis-KFL_FAK_ **(A)** or 6His-KFL_PYK2_ **(B)** were titrated against increasing concentrations of unlabelled KFL_FAK_ or KFL_PYK2_, respectively. Inlay shows the fluorescence scan of the capillaries. Figures were prepared using NanoTemper Analysis software (<https://nanotempertech.com>) **(C,D)** SEC-SAXS profile, showing the radius of gyration (*R_g_*) and the extrapolated intensity at zero angle (*I_0_*) for all collected images. *R_g_* and *I_0_* were derived from Guinier analysis (fit values are shown). **(E)** Thermal stability assay (DSF) showing the first derivative of melting curve. Figure was prepared using GraphPad Prism 6.0 (<https://graphpad.com>)


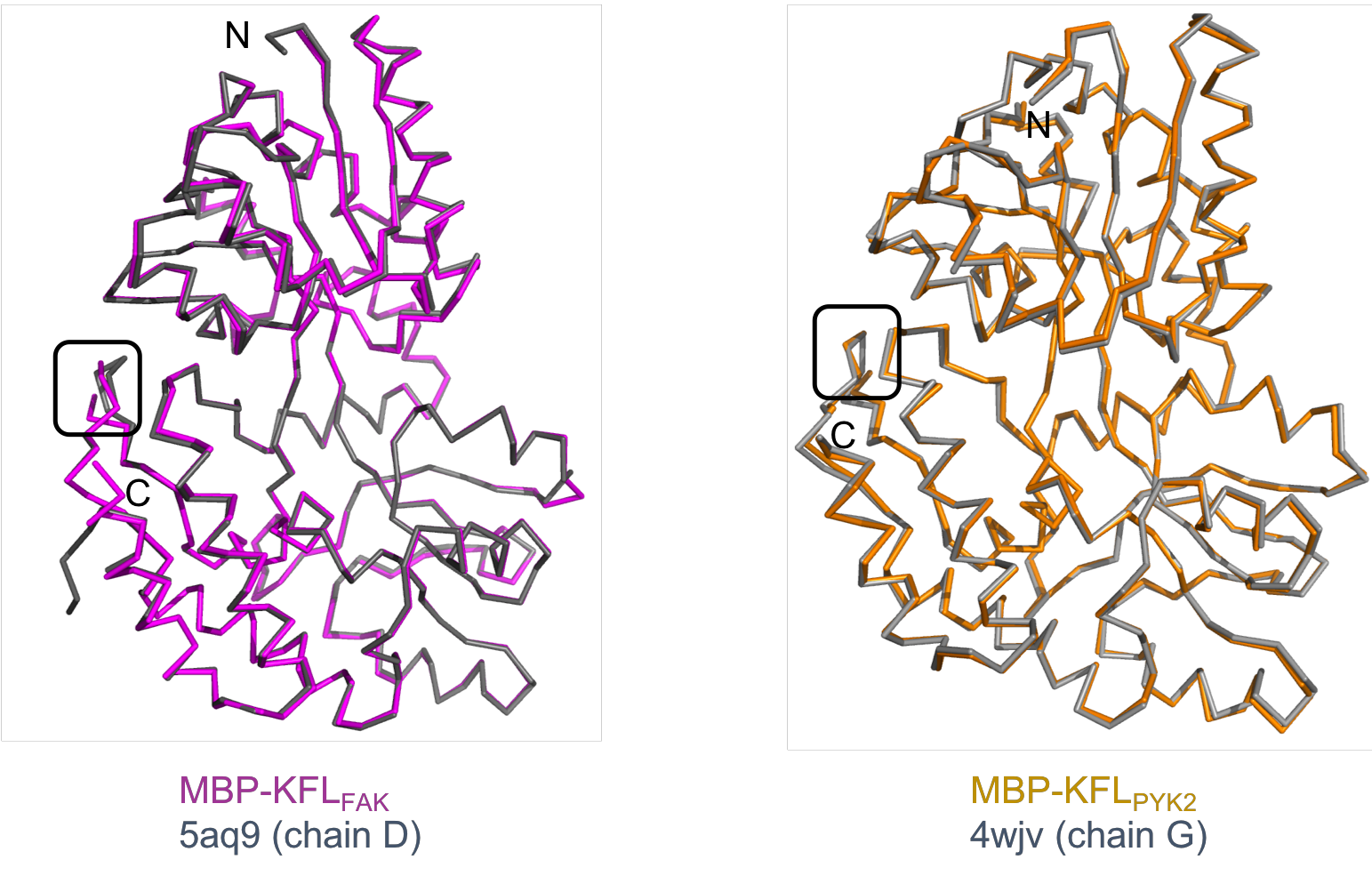


**Supplementary Figure 2:** Structural comparison of domain-swapped MBPeng (colored) with a monomeric MBP structure (grey). For this comparison, residues 3 to 174 of chain A were merged with residues 175 to 371 of chain B of a domain-swapped dimer to obtain a single structure. This structure was then used in PDBeFOLD to search for closest structural matches. Closest structural PDB accession number and chain ID are shown. RMSD FAK: 0.50 Å over 371 residues; PYK2: 0.42Å over 369 residues. Round-edged box indicates position of arm-exchange hinge loop; N and C termini are labelled. Figures were prepared using PyMol 1.8.6.2 (<https://pymol.org>)

**
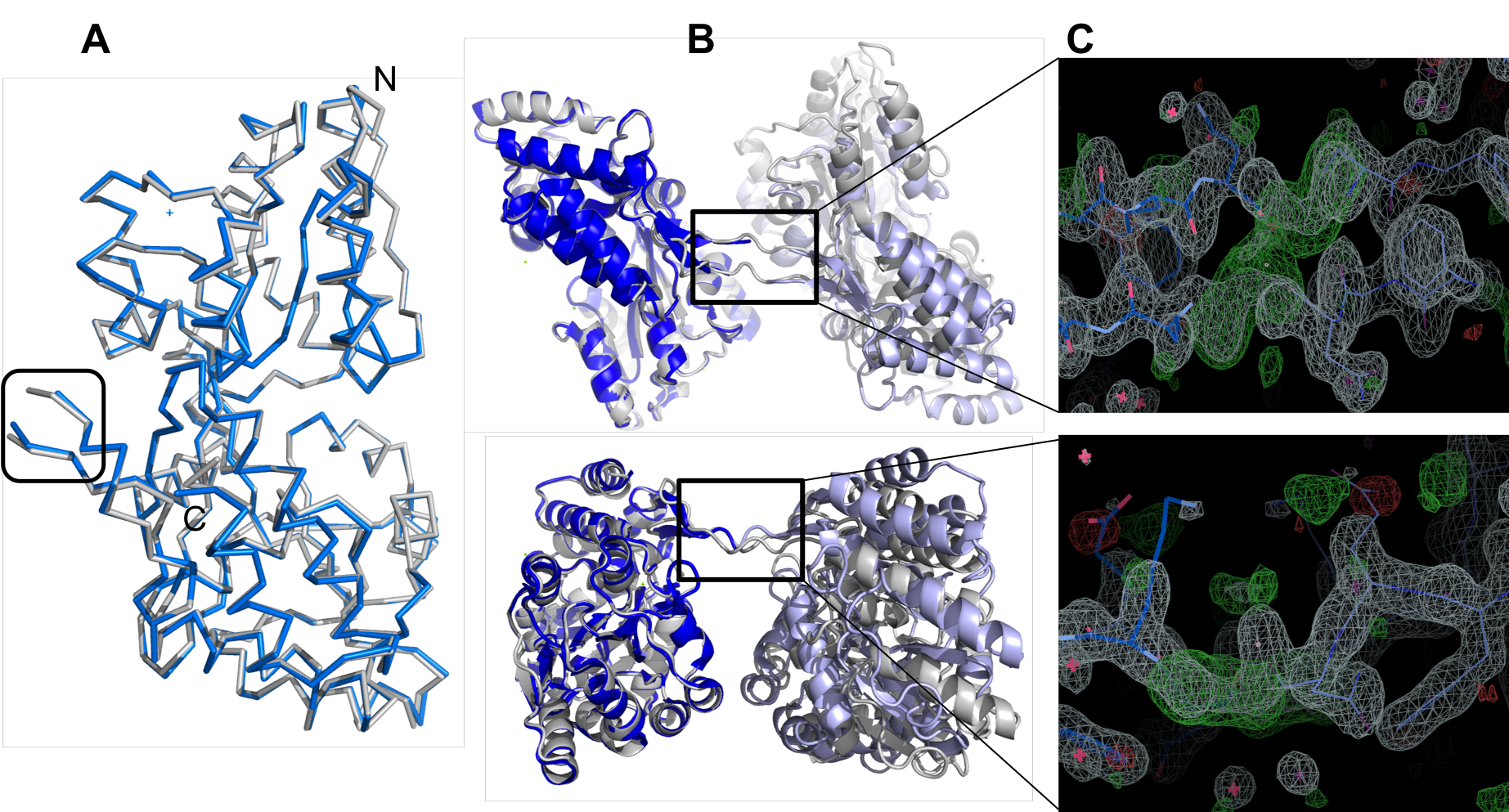
**

**Supplementary Figure 3:** Structural comparison of domain-swapped MBPeng (grey) with the structure of *Salmonella enterica* sugar binding protein MalE (PDB id 6l3e). **(A)** For this comparison, residues 3 to 174 of MBPeng chain A were merged with residues 175 to 371 of chain B of a domain-swapped dimer to obtain a single structure. The position of the hinge-region is boxed. **(B)** Superimposition of the domain-swapped dimer of MBPeng-KFL_PYK2_ (grey) with MalE (dark blue) and its corresponding crystal-symmetry related putative domain-swapped molecule (light blue). Top and bottom are 90˚ views. **(C)** Zoom into the boxed regions of **(B)**. 2FoFc map (grey, contoured at 1.2 σ) and FoFc maps (green, contoured at 2.8 σ and red, contoured at -2.8 σ) are superimposed onto the deposited PDB coordinates of MalE (right, dark blue) and its symmetry-related copy (light blue). Figures were prepared using PyMol 1.8.6.2 (<https://pymol.org>)


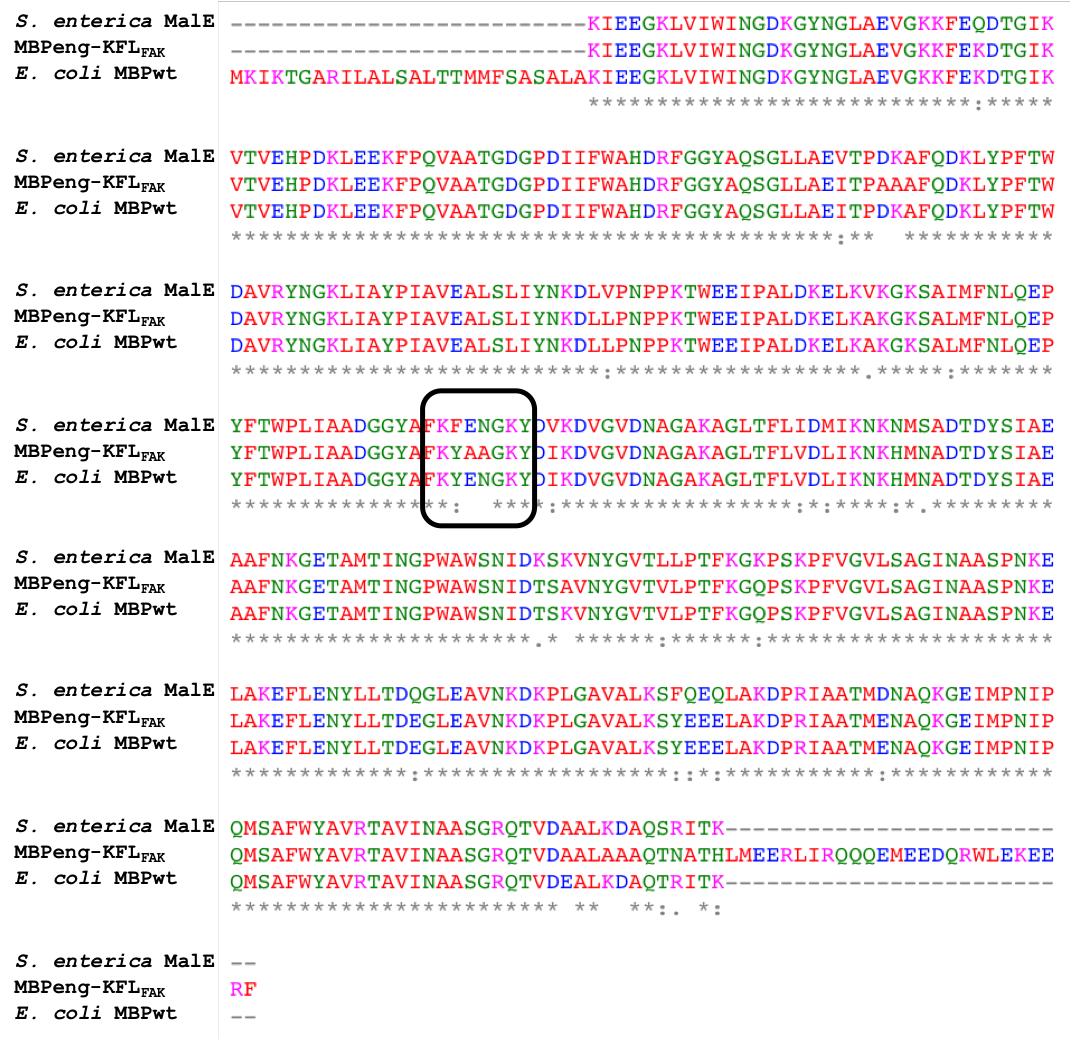


**Supplementary Figure 4:** Alignment of the sequences of MalE (PDB id 6l3e), MBPeng-KFL_FAK_ and E. coli MBPwt). The hinge region is boxed. Figure was prepared using Clustal Omega (<https://www.ebi.ac.uk/Tools/msa/clustalo/>)
